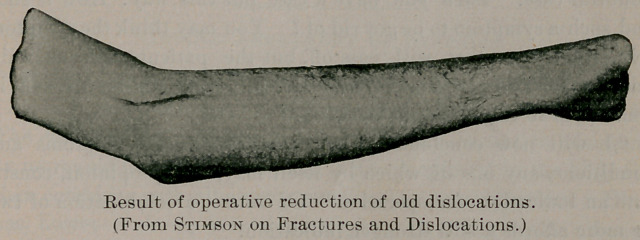# Selections and Abstracts

**Published:** 1899-03

**Authors:** 


### *SELECTIONS AND ABSTRACTS.*

### OLD DISLOCATIONS OF THE ELBOW.

Dislocations nowhere become inveterate and irreducible sooner than at the elbow. This is especially true in the young, where the developmental osteogenetic power of the periosteum is in full play, and where, consequently, the slightest injury or chronic irritation of the periosteum causes new bone-formation, the presence of which precludes the possibility of the joint surfaces resuming their old relations. The soft parts, too, in growing individuals are much more easily modified in their development by irritative factors than later in life, so that hindrance to the reduction of a dislocation soon supervenes in the course of a case from faulty evolution of the involved soft tissues. Finally, the ultimate bone relations in joints and the nice correspondence of opposing surfaces are the result of pressure and counterpressure of the parts upon each other during growth, and this being absent, deformity of the bony parts of the joints necessarily follows.

New formation of bone on an old, unreduced dislocation.

(From STIMSON on Fractures and Dislocations.)

The importance of the movements of the elbow-joint is very great, and, besides, from an esthetic standpoint, freedom of motion here is very desirable, since limitation of it always causes a striking peculiarity in the holding of the limb and awkwardness in the movement of it that are very noticeable. As stated before, reduction even by force soon becomes impossible. The necessity for early diagnosis and prompt reduction is greatly emphasized. Where inveteracy is once established, if the deformity is considerable, arthrotomy is indicated. The results of operative intervention have frequently in the past, however, been extremely unsatisfactory, and for two reasons: either too little of the abnormal structure that caused persistence of the dislocation were removed, in which case inevitably it recurred (often under the operation bandage); or too much of the bony structure was removed, an excision of the elbow being practically done, when a flail joint resulted—an eminently undesirable result.

Result of operative reduction of old dislocations.

(From Stimson on Fractures and Dislocations.)

Professor Stimson, in his new book on "Fractures and Dislocations," treats the subject with his well-known practical conservatism. He gives a sketch of a new formation of bone on an old, unreduced dislocation of the elbow, as he has seen it in a number of cases. He advises operation for the condition by a long incision on the outer side, exposing the radius and the mass of new bone. This should be freely chiseled away and the capitellum exposed by free division of the soft parts, keeping the knife at a little distance from the bone, so as not to damage the periosteum. The sigmoid fossa is then cleared of fibrous tissue. A second incision is now made on the inner side, curving close behind the epitrochlea or its site, the ulnar nerve is drawn forward,

Result of operative reduction of old dislocations.

(From STIMSON on Fractures and Dislocations.)

and the olecranon freed. If the epitrochlea has been broken off and displaced upward and backward it must be detached from the humerus, preserving its relations with the lateral ligament. The clearing of the sigmoid cavity is then completed. The only obstacle to reduction, then, if there be one, will be the shortening of the flexor muscles of the hand, induced by their action in the abnormal position caused by the dislocation. If necessary they must be partly divided close to the humerus. Professor Stimson gives a picture of one of his results, which we reproduce. Altogether he has operated upon some ten cases by this method, and the results have all been flexion within a right angle and extension varying from 120 to 170 degrees, with a preservation of rotation.

### INDICATIONS FOR OPERATION IN ADENOID DISEASE OF THE NASOPHARYNX.

Dr. James B. Ball (*Clinical Journal*, December 28th) concludes his paper as follows :

"Before laying down some general rules which guide one in advising operation or otherwise in adenoid cases, I may say that not only the cases, but the circumstances vary so much that no rules can cover every case. Thus with some patients there is such an excessive desire to avoid an operation, even at some risk, that this may naturally turn the scale in a doubtful case, and then you come across others who seem to be curiously keen to have their children operated on. Sometimes this is due to the exaggerated ideas that have got about on the serious effects of adenoids; but whatever it is due to, it may, of course, influence a decision in an otherwise doubtful case. Then you have a case put this way: How is such and such a symptom to be got rid of? You may think the symptom trivial; and one that will pass off, but the patients will not wait, and beg for an operation. These and other circumstances step in sometimes to influence our decision.

"I will now conclude by enumerating several symptoms and conditions, any one of which by itself may, in my opinion, constitute an indication for operation. Of course, a combination of two or more affords a still stronger indication.

"If there is habitual mouth-breathing in a child, which has been going on for a considerable period and shows no signs of improvement, I should operate. I should include also those children, especially young children, who, though not habitual mouth-breathers in the day, have noisy labored breathing or suffocative attacks at night. In such a case the appearance of a falling in of the lower part of the chest in a young child would considerably strengthen the indication.

"If the child is deaf, or subject to attacks of deafness or earache, or has a chronic otorrhea, I should recommend operation in all cases, and with a good prospect of cure; but in older children and young adults with long-standing deafness, though I still think we should operate, our prognosis must be guarded.

"Repeated attacks of bronchitis constitute an important indication; so does the presence of asthmatic symptoms. Distinct benefit may fairly be expected in these cases. A constant, persistent cough without bronchial symptoms, not yielding to ordinary treatment, is often cured by removal of adenoids.

"Repeated colds in the head of a severe and prolonged character, or a chronic nasal catarrh, or purulent rhinitis not yielding to simple treatment, may be an indication for operation. Paroxysmal sneezing and hay-fever symptoms are also indications for removal of adenoids.

"There are finally a few maladies, such as nocturnal enuresis, chorea, and epilepsy, where the operation my sometimes be done, although none of the foregoing indications are present, rather with a view to remove all possible sources of reflex irritation than with any distinct promise of direct benefit to the malady in question."

---

### IODINE TREATMENT OF SYPHILIS.

Zuelzer.—New propositions for the iodine treatment of syphilis. (*Arch. f. derm. u. syph. Festch. gewid. F. J. Pick,* 1898, p. 421.) The current view has been that after the administration of organic or inorganic iodine preparations, iodine is split off in the body and then combines with albumens, forming loose combinations. Blum has, however, shown that the halogens by acting upon albumen can form firm substitution products with elimination of hydriotic acid, and that this action can be proven in the thyroid after the administration of alkaline iodides, as here a storing up of these bodies occurs, while according to Zuelzer, proportionately little is found outside of the thyroid, and as in a short time outside of the thyroid no iodine is found, and as it is only eliminated in inorganic combination, therefore this organic compound must again be decomposed. Zuelzer studied the behavior of an iodine albumen compound in the organism, using iodalbacid, a preparation which contains 8 per cent iodine. This, unlike potassium iodide, is only oxidized in the test-tube by the strongest oxidizing agents, as sulphuric acid and potassium bichromate, yet the body oxidizes it, for after its administration alkaline iodides are found in the urine. It never causes iodism as does the potassium iodide, which the author believes due to the easy oxidizability of the latter. It is slowly absorbed and slowly eliminated, for while the potassium iodide may be eliminated in from three to four days, owing to its slow elimination, by the same amount of iodine in organic combination, the kidneys are only completely eliminated in four or five. In cases where the iodine salts could not be borne, the iodalbacid was taken with good results, and gastro-intestinal irritation, which is so common after the long use of the ordinary iodides, was entirely absent. In syphilis there has been a great variability in the use of the iodides; some use from 0.5--2 gm. *pro die*, while others run from 5.20 times the daily dose, and good results have been obtained with iodothyrin, which contains only a few milligrammes of iodine; at present one uses from 1—3 gm. and even this causes an oversaturation. The therapeutic value of this iodalbacid in syphilis is due to its slow, protracted action. In the secondary stage it is valuable. He proposes in the treatment of syphilis that during the first three or four years each mercury treatment (Herscheimer's method) should be followed by a three weeks' treatment with iodalbacid 3—4 gr. *pro die*, and if slight secondaries appear in the intervening period, iodalbacid should be given. During the tertiaries he recommends potassium iodide, followed by a six weeks' treatment of iodalbacid.—*The Dominion Medical Monthly.*

---

### THE EFFECTS OF THE HABITUAL USE OF ALCOHOL.

According to the *Dietetic and Hygienic Gazette* for January, Dr. Crothers of Hartford, Connecticut, in a paper read at the New York County Medical Association, October 17, 1898, discussed this subject in a very conservative and intelligent way. He stated among other things that the ingestion of alcohol accelerated the heart's action ten to fifteen beats a minute at first, but that after awhile the circulation became slower, so that the pulse-rate dropped at least as much as fifteen or twenty beats below the normal. Vision, he said, was always diminished and rendered unreliable by the ingestion of alcohol. The acuteness of the sense of hearing was likewise impaired. The increase in the pitch of the voice of persons under the influence of alcohol was due to impaired hearing. Hallucinations of hearing were frequent under such circumstances. The senses of taste and smell were also influenced by alcohol. The sense of touch was always exaggerated or diminished, and careful measurements of muscular force showed that the muscular system did not escape the deleterious action of this poison. If the temperate man suffered from the use of one or two ounces of alcohol in this appreciable way, then the continual or frequent drinking of alcohol must exert a distinctly deleterious influence. All experimenters agreed that when alcohol was taken in excess the result was a profound tissue and cell degeneration and general starvation of the tissues. Alcohol, by its anesthetic action, soothed the irritable nerve centers, and the effects were so pleasing that the individual desired to repeat the experience. Often this desire to seek relief by its anesthetic effect was really an indication of a diseased condition, which perhaps was disclosed only by a post-mortem examination. A century ago the anesthetic action of alcohol created dementia and idiocy, but to-day it was more apt to cause delirium and paralysis. Heredity seemed to be one of the most prominent causes of inebriety.

---

### PRESERVATION OF THE OVARIES IN GYNECOLOGICAL OPERATIONS.

Kelly.—Conservation of the ovary in hysterectomy and hysteromyomectomy. (*British Medical Journal*, 1898, v. 1, p. 288.) The change of name from "testes muliebus" to "ovaria" or "ovaries" has hindered the conservative treatment of female pelvic disorders, for had the older name "testes" been retained operative surgery would have advanced more slowly and would not have gone so far, as the conservatism with which the male organs are treated would have been reflected upon the gynecological field. The advantage of conservatism does not lie merely in the possibility of conception, but as Martin says, "it is probable that the ovaries, like the liver and thyroid gland, modify the blood circulating through them, and add to the blood some peculiar product of their metabolism. It may be that some of the climacteric symptoms are due to the loss of this substance from the system." Since 1895 Kelly endeavored to save the ovaries even in those cases in which it was necessary to remove the uterus and tubes; his chart shows a marked diminution and even absence of the nervous symptoms of the menopause. He thinks the operation most easily performed "by tying off the broad ligament at the uterine corner, including the isthmus of the tube and the utero-ovarian ligament in the first tie, tying the round ligament next, and then exposing the base of the broad ligament, ligating the uterine vessels, amputating the uterus at the vaginal junction, clamping the uterine vessels of the opposite side, and then pulling the uterus up and out, and ligating the round ligament, the tube, and the ovary at the opposite corner."—*The Dominion Medical Monthly.*

---

### Bicycle Riding and the Kidneys.

Miller.—Influence of bicycle riding upon the kidneys. A contribution to the knowledge of physiological albuminuria (*Muench med. Woch.*, 1896, No. 48). The urine of twelve healthy young bicyclists, between the ages of nineteen and thirty-two years, was examined. Eight of these were in training, and of these eight one had traces of albumen in his urine before training, while the urine of the remaining seven was normal. After the race albumen was present in seven out of the eight; in one there was only a trace, and in the remaining it was present in considerable quantity. Two cases, in one of which there was only a trace of albumen, contained a few hyaline casts, while the other six, including the one which had no albumen, contained as many casts as one usually meets in acute and chronic-parenchymatous nephritis. Most of the casts were hyaline, but there were also numerous granular and epithelial casts and some covered with renal cells. There were regularly present renal epithelium and a few leucocytes, but red corpuscles were absent. The albumen and casts disappeared in a few days. The urine of those not in training before the race contained no albumen, and after it albumen and casts were absent in two, in the third there was marked albuminuria, but no morphological elements, while in the fourth there was marked albuminuria with numerous casts. Leaving out of consideration the case in which albumen was present before the race, there was albuminuria in 72 per cent., a large number of casts in 58 per cent., a few hyaline in 15 per cent., and only 16 per cent. remained unchanged. This form of albuminuria due to bicycling is distinguished from the physiological albuminuria by its severity and the character of the casts. The author, however, believes that a persistent irritation of the kidneys is not to be feared. Nucleo-albumen, which is present in the urine after muscular exertion, could not be proved.—*The Dominion Medical Monthly.*

---

### TREATMENT OF BALDNESS BY SIMPLE ASEPTIC IRRITATION.

Jacquet (*La Presse Med.*, December 10, 1898) says that while all dermatologists agree that cutaneous irritation is the first principle in the treatment of baldness, there is no agreement as to the amount of irritation that will give the best result. Permanent irritation is certainly not as good as a slight irritation, which can be renewed at will. By this manner one gets the advantage of the vascular dilation, the hyperthermia, which favors the papillary vitality, and avoids the ultimate slowing of the blood stream, and the leucocytic migration and interstitial exudation which takes place if the irritation is continued to the point of inflammation. Transitory hyperemia can best be caused by repeated slapping of the scalp with a sharp brush, made of good pig's bristles. The brush should be applied all over the bald area, and along the margins of the hair. In a few seconds the scalp will become red and pulsating, a condition which will last for half an hour or more. The treatment should be repeated morning and night. In his own case Jacquet caused the hair to grow again on a bald spot in his beard as large as a two-franc piece, by making applications of the brush in the manner described twice a day for four months. In other cases he has made more frequent applications, five or six a day, and has attained more rapid results. To keep the brush in an aseptic condition, he plunges it each time, before it is used, into the following solution:

| | |
|---|---|
| Alcohol | ℥ iv. |
| Ol. ricini | ℥ i. |
| Hydrarg. chlor. corros | gr. ij. |
| Ext. opoponacis } aa | gtt. xxx. |
| Tinct. cocci cact. } aa | gtt. xxx. |

The brush is shaken as dry as possible before it is applied. If a brush is used which is made of wires, with a rubber back, it will be very easy to keep it aseptic by this solution.

### DRY TREATMENT OF SUPPURATIVE OTITIS MEDIA.

Goldstein (*Laryngoscope,* December, 1898) says that in most cases of suppurative inflammation of the middle ear, the dry treatment gives better results than treatment by irrigations and wet dressings. A tuft of cotton on a probe will absorb the secretion and cleanse the canal more effectually than a stream of fluid. When the membrane is perforated, as is usually the case, there is also the danger that a stream will convey the infection to a still healthy part, for instance, the mastoid cells. If the ear is treated dry, the formation of granulation tissue is also reduced to a minimum. If the opening in the membrane is very small, a Eustachian catheter may be used with a Globe nebulizer, containing a mixture of iodid 3 grains, carbolic acid 4 grains, and benzoinol 1 ounce. By steady inflation the remaining secretion may be forced out through the drum membrane, and at the same time an application of antiseptic substances is made to the mucous lining of the middle ear. A concentrated solution of peroxid of hydrogen is of advantage in seeking out pockets inaccessible to the syringe and the cotton-mop. A medicine-dropper is used for its introduction, and, by shifting the head of the patient, the peroxid can be made to come into contact with any portion of the middle ear which it is desired to reach. Emphasis is placed upon the necessity of keeping the nasopharynx in a clean and aseptic state in the treatment of suppurative otistis media.

---

### EPITHELIOMA OF THE PHARYNX.

Dr. Cecil E. Shaw (*Journal of Laryngology*, September, 1898) has reported a rare case of epithelioma of the pharynx. The patient was a fairly healthy married woman about thirty-eight years of age, with a good family history. In March, 1897, she began to feel some slight sore throat. By the end of April her voice became hoarse, and she began to experience difficulty in swallowing. There was no loss of flesh and no pain at any time.

On examination the pharynx was seen to be dry and dirty, and the laryngeal mirror revealed a swelling about the size of a nut on the posterior wall of the pharynx, directly behind the epiglottis, which it touched, and at the top the swelling was ulcerated at the point which came in contact with the epiglottis. No enlarged glands could be found in the throat or externally in the neck. Owing to the size and nature of the growth operative interference was out of the question. The patient gradually grew weaker, and died in July, five months after the growth was first noticed.

Malignant growths of the pharynx are rare, the least rare form being sarcoma of the tonsils, and no record was found of a malignant tumor on the posterior wall. McBride reports a case of epithelioma of the posterior pillars of the fauces in a woman, and Hill reports a similar growth in the glossoepiglottic fold in a man. Tilly recorded a tumor of the posterior wall of the pharynx just opposite the epiglottis, but there was a syphilitic history, and the tumor disappeared in a fortnight under the specific treatment, while in Dr. Shaw's case specific treatment had no effect.

---

### REBELLIOUS CONSTIPATION CURED BY MASSAGE OF THE GALL-BLADDER.

Berne (*Jour. des Practiciens*, No. 47, 1898) has found that in certain patients massage of the gall-bladder gives almost as good results in overcoming constipation as massage of the whole abdomen. In some patients massage of the whole abdomen is contraindicated, as in the presence of an abdominal tumor, or an excess of adipose tissue, or a particularly sensitive skin, as well as in young women in whom the close association of the pelvic and abdominal organs make general abdominal massage undesirable. Moreover, constipation is often due to sluggishness of the biliary flow, and massage over the gall-bladder corrects this. The operator sits at the right side of his patient, and makes deep, gentle plunges with his fingers from below the false ribs upward toward the under surface of the liver. Ten minutes of this exercise, three hours after the midday meal, and repeated daily for ten or twelve days, will suffice to bring about a normal passage without the help of drugs. From thirty to forty days are required to effect a permanent cure. The passage of bile into the intestine is shown by the restoration of the normal color of the stools, and the disappearance of their fetid odor, while the body of the patient regains its natural embonpoint.

---

### TREATMENT OF VARICOCELE BY SUBCUTANEOUS LIGATURE.

Nimier (*Rev. de Chir.*, October, 1898) suggests the following simple method of treating a varicocele: The patient lies upon his back and the testicles are held well up against the pubic bone by an assistant, the redundant scrotum being spread out like an apron before the thighs. A Reverdian needle is thrust into the scrotum from the left side, and passes across its whole breadth, just beneath the skin anteriorly until it emerges at the right side. It is threaded with silk and withdrawn. It is then introduced into the same puncture on the left side, and passes along the posterier wall until it emerges through the puncture at the right side. Here the other end of the silk thread is passed through its eye and the needle is again withdrawn. The silk thread then surrounds the whole scrotum subcutaneously. It is drawn tight and tied, the knot being pushed through the opening in the skin. A little cotton and a suspensory bandage constitute the dressing. The reaction is slight, but it is better for the patient to remain in bed for one week. The writer insists upon the importance of strict asepsis.

---

### THE TOPICAL USE OF QUININE IN LEUCORRHEA.

W. W. Hardwicke, in *The Lancet* of January 7, 1899, says that some time ago he accidentally learned that quinine was a valuable agent in this affection, and has since used quinine topically in several cases of simple leucorrhea, and in every case with great success; in fact he does not know of a single instance in which it has failed, or in which quininism has been produced. It may be used in the form of douche or suppository. He adopts the latter form as being obviously the better one, the drug having a better chance of closer and more continuous contact with the congested membrane, and prescribes three grains of hydrobromate in a half-drachm suppository in combination with oleum theobromatis; but the pessus quininæ of the "Extra Pharmacopœia," containing the hydrochloride, answers just as well. One insertion a day is generally sufficient, a good result being very soon manifest.

It is a matter of astonishment that quinine, in the form of pessary, has never been used before in the treatment of leucorrhea and ulceration, for its valuable properties—tonic, astringent, and antipyretic—suggest it as a useful remedy in such cases, but the writer can find no record of such in any of the well-known works on the subject.

---

### THE EFFICACY OF GUAIACOL IN THE TREATMENT OF EPIDIDYMITIS.

J. C. Perry, in the *Medical Record* of January 7, 1899, in using guaiacol in the treatment of epididymitis, says his method is to apply one cubic centimeter of pure guaiacol over the cord of the affected side as it lies in the inguinal canal, and to paint the scrotum over the inflamed epididymis with two cubic centimeters of a mixture containing one part of guaiacol and two of glycerin. This, says the author, does not produce much burning, and although some peeling of the skin of the scrotum occurs, it seldom causes discomfort. Two applications are made during the day—one in the morning, the other in the evening—but if the attack is very severe, a third is used on the first day of treatment. The writer's experience has taught him that four or five applications are sufficient to effect a cure in the same number of days, and that a liberal use of this agent will often abort an attack of epididymitis when it is seen in its commencement.

In the chronic form of the trouble, guaiacol has no appreciable effect.

---

### THE EFFECT OF GLAUBER SALT UPON THE STOMACH FUNCTIONS.

Simons (*Zeitschrift für Klinische Medicin*, Vol. XXXV., No. 3, u. 4), having systematically studied the effect of Glauber salt in cases of stomach ailments, finds that sodium sulphate in daily doses of fifteen grains, produces a stimulating effect on the stomach and intestinal peristalsis, which is preceded by such symptoms as restlessness in the abdomen, borborygmus, etc. Its administration is very favorable in cases of anacidity and mucous catarrh, but is not usually to be recommended in cases of atrophic stomach catarrh, systemic anacidity, dilatations, and lesions of motility. No result occurred in the treatment of nervous indigestion without local changes, or hyperacidity. The chemical qualities of the gastric juices were only affected in cases of gastritis, and then in the form of increased acidity. In no case had diminution of acidity been noticed. The author remarks, in conclusion, that the analogy between sodium sulphate, the Carlsbad Thermalwasser, and the Carlsbad Quelle salts is evident, only that in antithesis to these latter it does not reduce the hyperacidity of the gastric juices.

---

### FETID BREATH AND ITS TREATMENT BY CARBONATE OF CREOSOTE.

Bayer (*Rev. de Therap.*, December 15, 1898) believes that chronic affections of the nasopharynx are the cause of fetid breath in obstinate cases. For instance, in women who are annoyed by bad breath at the period of menstruation, one can always make out at such times a congestion of the nasopharynx with an increase of its secretion. The best treatment is the intratracheal injection of carbonate of creosote. It is better to use the carbonate than the pure creosote, as the latter sometimes provokes spasms of suffocation, whereas the carbonate of creosote, slightly warmed, may be injected by the ordinary intralaryngeal syringe without unpleasant effects, in quantities of .5 to 1.5 c.c. (15 to 45 drops). Such an injection does not produce irritation, and the relief is often so marked that there is no occasion to repeat it.

---

### TUBERCULOSIS OF THE KIDNEY.

Friedjung, in the *Archiv für Kinderheilkunde*, Bd. 22, Heft 5 u. 6, reports an interesting autopsy where in addition to tuberculosis of the spine, the disease had invaded the kidneys and ureters. This is acccounted for by the child having had scarlet fever with nephritis two years previously, leaving the kidneys predisposed for tuberculosis.

## Figures and Tables

**Figure f1:**
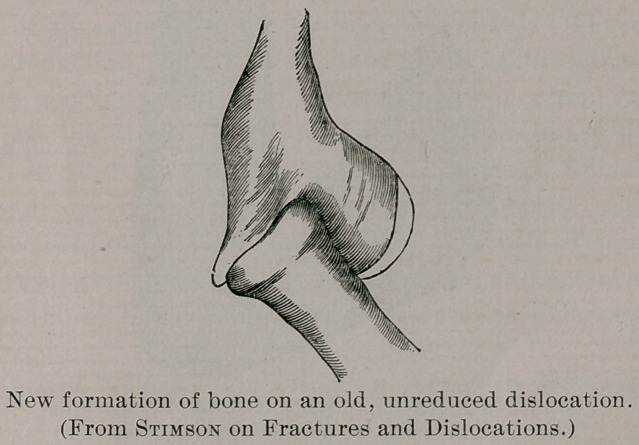


**Figure f2:**
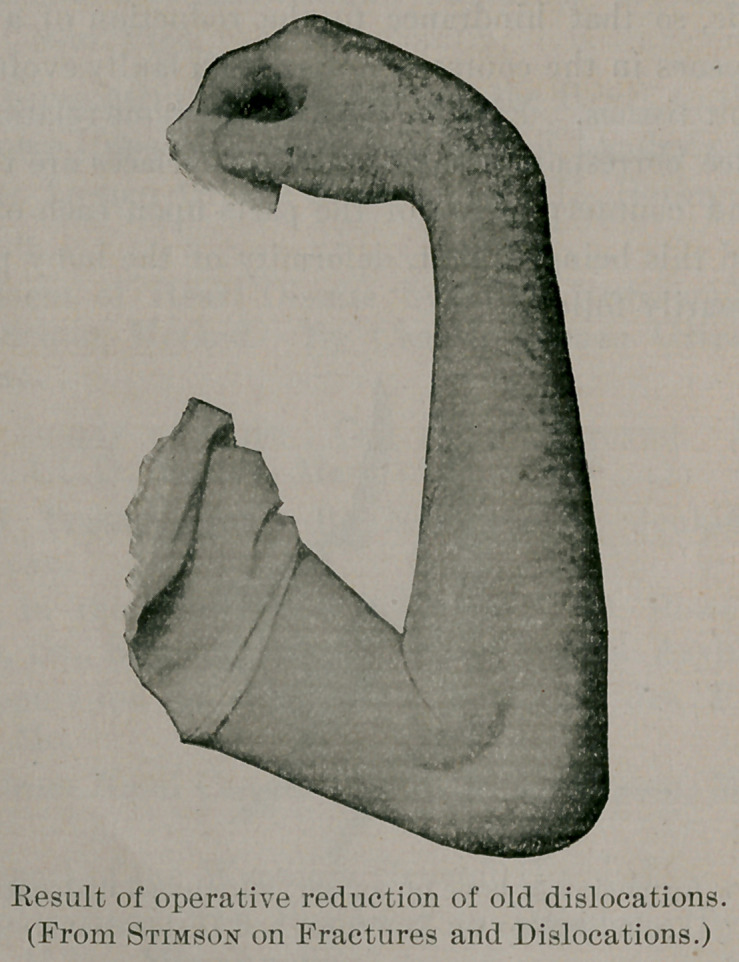


**Figure f3:**